# Use and Perception of Video Consultations Among Swedish Dietitians Before and After COVID‐19 Onset

**DOI:** 10.1111/jhn.70080

**Published:** 2025-06-18

**Authors:** Sarah Persson, Anette Edin Liljegren, Cecilia Olsson, Petra Rydén

**Affiliations:** ^1^ Department of Food, Nutrition and Culinary Science Umeå University Umeå Sweden; ^2^ Department of Epidemiology and Global Health Umeå University, The Swedish Centre for Rural Health, Region Västerbotten Storuman Sweden

**Keywords:** dietetics, digital health, distance counselling, health services accessibility, telehealth, telenutrition

## Abstract

**Introduction:**

The implementation of telehealth began globally before the onset of COVID‐19 but the use of telehealth, particularly video consultations (VCs), is expected to have increased with pandemic restrictions on face‐to‐face consultations (FTFCs). However, little is known about its actual usage. In Sweden, VCs have the potential to bridge long distances between Registered Dietitians (RDs) and their patients. This study investigates the use and perceptions of VCs among Swedish RDs before and after the onset of COVID‐19.

**Methods:**

Swedish RDs were invited to participate in web‐based surveys in 2016 (*n* = 61) and 2021 (*n* = 112). Data are analysed and later discussed through the lens of Levesque et al.'s framework for patient‐centred access to healthcare.

**Results:**

More RDs reported having VC‐experience in 2021 compared to the 2016 survey, 67% and 16% respectively. A majority of the RDs (85%–88%) believed that access to dietetic care would increase with the use of VCs compared to FTFCs. In 2021, about half of RDs (55% and 46%) perceived treatment quality and relational quality to be unaffected by VCs, while approximately one‐third (31% and 43%) saw it as being reduced. With their additional experience, there was the caution by 69% of RDs in 2021 that consultations requiring language interpretation services were less suitable for VCs.

**Conclusions:**

The findings suggest broader VC usage among Swedish RDs participating in the study. Implications for clinical practice include maintained *access* to healthcare and further practice development to meet quality needs and increased equity.

## Summary

- Video consultations were perceived to increase access to healthcare compared to face‐to‐face consultations.
- Treatment quality and the therapeutic relationship were thought to be unaffected by some, and reduced by others, with video consultation use compared to a face‐to‐face consultation format.
- Video consultations were perceived as less suitable for patient consultations requiring language interpreters than other consultation situations.

## 1 Introduction

Worldwide, there are challenges in providing accessible, cost‐effective, environmentally‐friendly, and high‐quality healthcare services equally to all citizens [1, 2]. Using information and communication technology (ICT) to provide healthcare remotely is defined as telehealth [3] (e.g., video and telephone consultations). This may increase access to treatment compared with face‐to‐face consultations (FTFCs) with its geographical flexibility, potentially reducing inequalities [1, 3, 4, 5, 6]. Additionally, by reducing the need to travel, telehealth may offer environmental benefits and cost savings [2, 7, 8]. Telehealth has evolved rapidly in the 21st century [3], and the use of video consultations (VCs) has grown as mobile technology and high‐speed internet became publicly available [9]. Before the COVID‐19 pandemic, VC‐inexperienced patients and healthcare professionals were skeptical of VC usage [10, 11]. Many VC‐projects failed to reach permanent implementation [12, 13], although they worked well in crises [14, 15, 16]. Compared to FTFCs, known barriers to VCs include access for different groups, technical limitations, and digital literacy [3, 17, 18, 19]. Problems establishing a therapeutic relationship and ensuring treatment quality compared with FTFCs also raise questions about VC appropriateness for first‐time consultations [20, 21]. With the pandemic onset in early 2020, social distancing restrictions came into force, and telehealth became the primary mode of healthcare worldwide [12]. Consequently, the use of VCs within healthcare is expected to have increased. To evaluate the effect of VCs more data is needed about use.

Access to healthcare is defined as the opportunity to reach and obtain appropriate healthcare services in situations of perceived need for care [22]. According to Levesque et al., there are five essential dimensions to healthcare access [22]. Firstly, the *approachability* of healthcare, such as the consultations offered, is related to the patient's ability to *perceive* the opportunity for care. Secondly, the *acceptability* of the provided care within healthcare is related to the patient's ability to *seek* care. Thirdly, the *availability* of healthcare affects the patient's ability to *reach* care. Fourthly, *affordability* concerns the costs for healthcare and the patient's ability to *pay* for care. Lastly, the *appropriateness* of healthcare relates to the patient's ability to *engage* with the healthcare provided. Video consultations have the potential to address these dimensions, but further investigation is needed to determine how each dimension interacts with VCs to improve access to healthcare.

In Sweden, access to dietetic treatment is limited due to the low number of Registered Dietitians (RDs) per capita, recruitment difficulties, and centralized dietetic units, leaving large areas without nearby RD‐services [23]. Most dietetic treatment occurs within the public healthcare system, either in primary care (outpatient setting) or at a hospital (in‐ and outpatient setting), with few additional private RD practices. In 2016, VCs was mainly provided for remote patients [24, 25] and Sweden adopted a vision for broad eHealth implementation [26]. The first of 21 healthcare regions launched a telehealth mobile app for VCs [27]. During the pandemic, social distancing was strongly recommended but lockdowns were not enforced [28]. Most healthcare regions in Sweden still allowed FTFCs with patients if telehealth was not an option, but a large increase in the use of VCs was seen in primary care [29]. By 2021, telehealth apps were implemented in 18 out of 21 regions, and 79% of Internet users in Sweden used digital healthcare services [30].

Telenutrition, defined by the Global TeleNutrition Consortium [31] as “the use of different modes of communication to deliver nutrition care service at a distance,” applies the WHO's definition of telemedicine [1] to nutritional care. Telehealth can improve nutritional care by enhancing access and effectiveness [32, 33, 34, 35]. Registered Dietitians (RDs) typically deliver nutritional care by following the Nutritional Care Process where Nutrition Assessment and Reassessment of body composition and dietary intake are essential in addition to Nutrition Intervention, and Nutrition Monitoring and Evaluation [36]. Telenutrition challenges traditional Nutrition Assessment and Reassessment methods, but modified practices are suggested for data collection [31, 37, 38]. During pandemic restrictions, telenutrition enabled continued care [39]. Nevertheless, little is known about how the use of VCs was affected by the pandemic. Few studies have investigated telenutrition use before and after COVID‐19 onset [40, 41]. They show increased use of telenutrition as an effect of pandemic restrictions, but their pre‐pandemic data was retrospectively collected. Thus, further research is needed to develop a better understanding of the pandemic's impact on nutritional care. The Dietitian Online (DiOn) project by The Swedish Centre for Rural Health and Umeå University aimed to evaluate VCs' effect on patients, RDs, and society [42, 43]. The aim of this study was to investigate the use and perceived appropriateness of VCs in the professional practice of clinically‐active Swedish RDs before and after the COVID‐19 onset in March 2020.

## 2 Materials and Methods

### 2.1 Study Design

This study had a cross‐sectional design. Two online surveys were conducted among Swedish RDs, one in 2016 and another in 2021, using nonprobability sampling [44].

### 2.2 Survey 2016

The first survey was conducted in October 2016 (Survey 2016). This was constructed using the Google Forms survey tool and consisted of 33 questions. It investigated the anticipated impact of VC use on professional practice compared to FTFCs, VC suitability for different patient groups, demographic data, VC access, and experience with VCs. The survey was piloted among RDs within academia, some with previous clinical experience. However, the survey questions were not otherwise validated.

**Figure 1:**
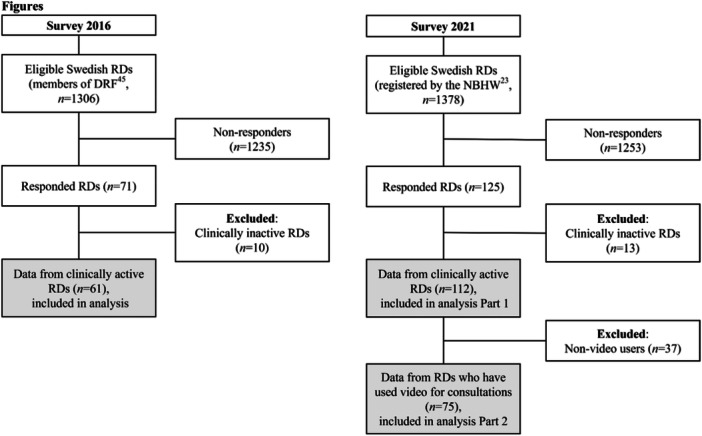
Flowchart of recruitment, exclusion, and inclusion for analysis in the study. DRF = Swedish Association of Registered Dietitians, NBHW = The National Board of Health and Welfare.

The survey was distributed through the Swedish Association of Registered Dietitians (DRF) electronic newsletter to all their members via email, with one reminder, and was open for 2 weeks. All members of DRF (*n* = 1306) [45], representing approximately 70% of RDs in Sweden, were eligible to participate in the survey. Clinically‐inactive RDs were excluded from this analysis as the aim was to study RDs working with patient treatment (Figure 1). Clinically‐inactive RDs were RD students, and RDs engaged only with administrative work without any patient consultations during the same year of the study.

The survey consisted of single‐choice and Likert scale questions—most with the opportunity to write comments—as well as open‐ended questions. The Likert scale choices were developed to capture perceptions ranging from negative to positive. The questions regarding the perceived suitability of VCs for first‐time and follow‐up consultations were designed to ask for positive perceptions only (no benefits to high benefits). This approach aimed to capture more stratified responses and identify potential differences between the two types of consultations during treatment.

### 2.3 Survey 2021

The second survey was conducted between November 2021 and January 2022 (Survey 2021), when pandemic restrictions had been in place for 1.5 years in Sweden. The survey was designed to investigate the development of VC use and its impact on professional practice. This consisted of two parts and contained 29 questions using the survey tool Survey & Report from Artologik. The first part consisted of demographic questions. The second part included questions about professional practice with VCs in relation to FTFCs and telephone consultations (TCs), VC suitability for different treatment situations, and RD thoughts and wishes for the future. The survey questions were developed based on the experiences from the 2016 survey; however, they were not further validated.

The survey was distributed through the DRF electronic newsletter to all members via email, with one reminder. The survey was open for 12 weeks. In 2021, DRF had approximately 1300 members. Only clinically active RDs were invited to participate in the survey. The survey was also distributed through two private Facebook groups dedicated to RDs in Sweden: DRF's Facebook group with 1250 members (available only to members) and a private Facebook group (Dietisterna) with almost 1200 members. Two reminders were posted in each Facebook group. For RDs reporting not having used VCs in the last year, only the first part of the survey was available (Figure 1).

The survey consisted of single‐choice, multiple‐choice, and Likert scale questions—some with the opportunity to add comments—as well as open‐ended questions. The Likert scale choices were developed to capture perceptions ranging from negative to positive. Answer alternatives for questions regarding the perceived suitability of VCs in different settings were designed with the binary—“most suitable” and “least suitable”—to capture potential patterns in perceptions across different situations.

### 2.4 Data Analysis

The Likert scales used in the 2016 survey were regrouped for analysis as follows: For the 11‐step scales; much decreased (0–1), decreased (2–4), unchanged (5), increased (6–8), much increased (9–10). For the 10‐step scales; very low confidence (1–2), low confidence (3–5), confident (6–8), high confidence (9–10); and no benefits (1–2), low benefits (3–5), some benefits (6–8), high benefits (9–10). The Likert scales used in the 2021 survey were regrouped as follows: For the 7‐step scales; much decreased (1), decreased (2–3), unchanged (4), increased (5–6), much increased (7).

Comparisons between groups were performed using the *χ* ^2^ test and the Mann–Whitney *U*‐test for non‐parametric data. Within‐group comparisons were performed using Wilcoxon Signed Ranked test and Friedman's ANOVA. Data were analysed using IBM SPSS Statistics (version 28.0.1.1 (15)). A significance level of 0.05 was used.

The conceptual framework of Levesque et al. for patient‐centred access to health care [22] was applied when interpreting the results in discussion together with previous research. Four of the five dimensions of the framework were used to understand the implementation process of VCs.

### 2.5 Ethical Considerations

The study follows the Declaration of Helsinki and has been approved by the Swedish Ethical Review Authority (dnr 2015/330‐31). Participation was anonymous and voluntary, and no compensation was offered. Information about the study and the opportunity to ask questions to the research group was provided in a written letter of consent at the beginning of each survey. Consent was given by submitting the survey.

## 3 Results

### 3.1 Participants

Survey 2016 received 71 responses. After excluding clinically‐inactive RDs (*n* = 10), 61 responses remained, corresponding to 5% of the clinically‐active RD population (*n* = 1205) in Sweden [23] (Figure 1). Survey 2021 received 112 responses, corresponding to 8% of the clinically‐active RD population in Sweden (*n* = 1378) [23]. Participants not using VCs for the last year (*n* = 37) were excluded from the second part of the survey, 75 responses remained (Figure 1).

Characteristics of participants are provided in Table 1. Commonly, they worked within a hospital setting (64%, *n* = 111) and had worked as RDs for 6–15 years (35%, *n* = 60). Slightly more than half of them were younger than 45 years in Survey 2016 (54%, *n* = 33) whilst almost two‐thirds were < 45 years in Survey 2021 (63%, *n* = 71). The RDs represented 16 (Survey 2016) and 17 (Survey 2021) out of 21 healthcare regions, with the three regions inhabited by half of the Swedish population [46] represented by 38% of the RDs (*n* = 23) in Survey 2016, and 44% (*n* = 49) in Survey 2021. The proportion of RDs travelling for patient consultations was reported as 25% (*n* = 15) in the 2016 survey and 20% (*n* = 20) in the 2021 survey. Data on gender are not reported due to the very small population of male RDs in Sweden.

**Table 1:** Characteristics of Swedish registered dietitians participating in the study. Differences between the surveys analysed with Pearson's *χ* ^2^ test.

Participant characteristics	Survey 2016		Survey 2021		*p*
	(*n* = 61)		(*n* = 112)		
	*n* (%)		*n* (%)		
Workplace					0.632
Hospital	40 (66)		71 (63)		
Primary care	15 (25)		32 (29)		
Other	6 (10)		9 (8)		
Working experience (years)					0.078
0–2	5 (8)		16 (14)		
3–5	4 (7)		19 (17)		
6–15	23 (38)		37 (33)		
16–25	17 (28)		27 (24)		
> 25	12 (20)		13 (12)		
Age (years)					0.121
20–34	18 (30)		42 (38)		
35–49	22 (36)		43 (38)		
50–64	18 (30)		27 (24)		
> 64	3 (5)		0 (0)		
Work region	*n* = 60				0.859
* n* of regions/total *n* in Sweden	16/21		17/21		
North	15 (25)		29 (26)		
Central	20 (33)		41 (37)		
South	25 (42)		42 (38)		
Travels to meet patients	*n* = 59				
Yes	15 (25)		22 (20)		0.383
Experience/use of video meeting					
Yes	10 (16)		75 (67)		< 0.001

### 3.2 Usage of VCs

Two‐thirds (67%, *n* = 75) of the RDs had conducted VCs with patients during the last year in Survey 2021, compared with 16% (*n* = 10) in Survey 2016 who had any experience of VCs in their clinical setting (*p* < 0.001, Table 1). In both 2016 and 2021, RDs believed they would use VCs more frequently in the upcoming year.

In 2021, 16% (*n* = 12) of the RDs had daily VCs. Almost half of them (49%, *n* = 37) used it weekly, about one‐fourth (24%, *n* = 18) at least once a month, and one in ten (11%, *n* = 8) used it less often than once a month. RDs working in primary care (*n* = 27) had more VCs than RDs working in hospital care (*n* = 41, *p* = 0.001). Of those using VCs in Survey 2021, 75% (*n* = 55) began using VCs in March 2020 or later (pandemic adopters). The pre‐pandemic adopters were significantly fewer (*n* = 18, 25%, *p* < 0.001), met patients from larger catchment areas (83% vs. 53%, *p* = 0.021), and used VCs more frequently (weekly or daily, 83% vs. 58%, *p* = 0.010) than the pandemic adopters. Almost all RDs using VCs in 2021 (89%, *n* = 67) had a positive attitude toward VCs in clinical practice (Table 2).

**Table 2:** Dietitians' anticipation of how video consultations will affect clinical practice, compared to face‐to‐face consultations, and their assessment of confidence in video consultation practice (Survey 2016). Additionally, estimations of the effect of video consultations in clinical practice among dietitians experienced with video consultations compared to face‐to‐face consultations (Survey 2021); and their overall attitude towards video as a consultation modality.

	Survey 2016 (*n* = 61)						Survey 2021 (*n* = 75)				
	*n* (%)	*n* (%)	*n* (%)	*n* (%)	*n* (%)		*n* (%)	*n* (%)	*n* (%)	*n* (%)	*n* (%)
	Much decreased	Decreased	Unchanged	Increased	Much increased		Much decreased	Decreased	Unchanged	Increased	Much increased
*Access to care*	0 (0)	0 (0)	11 (18)	26 (43)	24 (39)		0 (0)	1 (1)	8 (11)	36 (48)	30 (40)
*Quality of relationship with patient*	2 (3)	26 (43)	28 (46)	5 (8)	0 (0)		1 (1)	27 (36)	39 (52)	6 (8)	2 (3)
	—	—	—	—	—	*Treatment quality*	0 (0)	23 (31)	41 (55)	9 (12)	2 (3)
*Compliance with treatment*	0 (0)	15 (25)	32 (53)	11 (18)	3 (5)		—	—	—	—	—
	—	—	—	—	—	*Physical working environment*	0 (0)	6 (8)	45 (60)	16 (21)	8 (11)
	—	—	—	—	—	*Psychosocial working environment*	1 (1)	11 (15)	43 (57)	14 (19)	6 (8)
	No benefits	Low benefits	Some benefits	High benefits							
*First‐time consultation*	13 (21)	36 (59)	10 (17)	2 (3)			—	—	—	—	—
*Follow‐up consultation(s)*	2 (3)	17 (28)	21 (35)	21 (35)			—	—	—	—	—
	Very low confidence	Low confidence	Confident	High confidence							
*Confidence in video consultation practice*	0 (0)	16 (26)	29 (48)	16 (26)			—	—	—	—	—
							Negative	Neutral	Positive		
	—	—	—	—		*Attitude to video consultation*	1 (1)	7 (9)	67 (89)		

In 2016, approximately one‐third (36%, *n* = 22) of the RDs had access to VC‐equipment in their healthcare unit, but only 16% had used it. Nevertheless, many (74%) expressed confidence in managing VCs with their patients (Table 2). Access to appropriate technical equipment, approved software, education, training, and the patient's ability to manage technical requirements were stated as prerequisites for implementing VCs.

In Survey 2021, the decision of consultation modality (VC, TC or FTFC) for patient consultations involved three actors. For first‐time consultations, the RDs commonly decided on the modality to be used (56%, *n* = 42). About one‐third were decided by the patient (29%, *n* = 22), and 12% (*n* = 9) by the healthcare organisation. For the follow‐up consultations, about equally half of the consultation modality decisions were made by the RD or the patient, 47% (*n* = 35) and 45% (*n* = 34) respectively. For both consultations, the remaining percentages represented “Other options”, specified in comments as variations involving all three actors dependent on context. In open text responses, many RDs wished to clarify that the consultation modality choice often was a collective decision between the patient and RD (above counted as patient‐decided).

**Figure 2:**
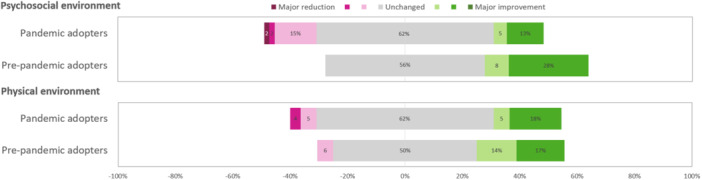
Consultation modality choice, graded on a 5‐grade Likert scale from Never to Always, for first‐time and follow‐up consultations with patients by dietitians with experience of video consultations in Survey 2021. Differences in consultation modality within first‐time consultation and follow‐up consultations analysed using Friedman's ANOVA (*n* = 73–76). Differences in consultation modality between first‐time consultation and follow‐up consultations; Face‐to‐face *p* = 0,003, Video *p* = 0,121, Telephone *p* < 0,001, analysed using Wilcoxon Signed Ranked test (*n* = 70–71).

In Survey 2016, 70% of the RDs believed that VCs would be more suitable for follow‐up consultations with patients. Most RDs (80%) predicted that VCs would have few to no benefits as consultation modality for their first‐time consultation with a patient (Table 2). In open‐text responses, RDs favored initial FTFCs for building rapport (thus facilitating an improvement in treatment outcomes), taking measurements, assessing nutritional status, and using visual aids. Video consultations were considered an acceptable first‐time alternative if FTFCs were considered impractical (due to delays/patient barriers) and advantageous for follow‐ups once rapport was established by FTFC. In Survey 2021, VCs was equally used as a consultation modality for first‐time and follow‐up consultations (*p* = 0.121, Figure 2). Video consultations were also as equally used for first‐time consultations as FTFCs (*p* = 1.000) and TCs (*p* = 0.443). Also, for follow‐up consultations, no differences could be seen between the three consultation modalities (*p* = 0.668). Face‐to‐face consultations were more commonly used for first‐time consultations compared with follow‐up consultations (*p* = 0.003) and TCs were more common for follow‐up consultations rather than first‐time consultations (*p* < 0.001).

**Figure 3:**
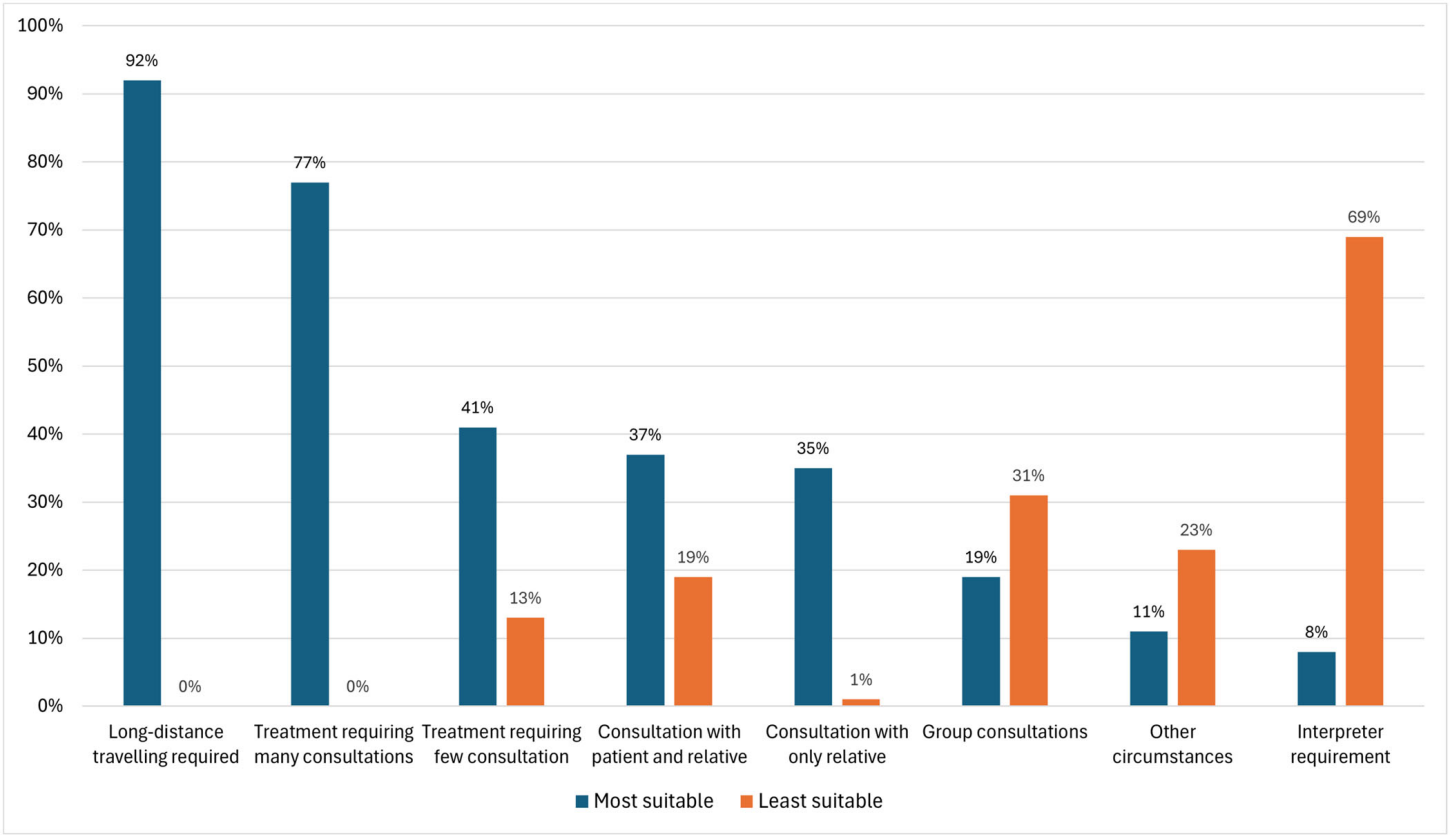
Perceived suitability of video as consultation modality, rated by dietitians with experience of video consultations in Survey 2021 (*n* = 75). Multiple answers possible.

In Survey 2021, VCs were perceived to be most suitable when FTFCs would require extensive travelling (92%, *n* = 69), when multiple sessions are needed (77%, *n* = 58), or when the consultation was solely with relatives (35%, *n* = 26, Figure 3). Consultations exclusively intended to provide information, or when patients experienced difficulties with mobility or preferred not to receive FTFCs were also described as suitable for VCs. Situations where VCs were described as least suitable included sessions with patients in need of language interpreter (69%, *n* = 51, Figure 3), had cognitive impairment, when anthropometric measurements were needed, when conversations turned emotional, when RDs wanted to discuss visual aids with patients, or when there was a lack of digital literacy for the patient or RD. Group consultations, consultations including patients and relatives, and treatments involving few sessions were described both as suitable, as well as less suitable, for VCs by different RDs (Figure 3). In Survey 2016, many RDs expressed opinions about certain groups of patients that they believed VCs would either suit or be unsuited to, such as age and disease‐related conditions. However, no distinct pattern emerged.

Video consultations were anticipated to increase access to healthcare by 82% of the RDs in Survey 2016, compared to FTFCs (Table 2). Reduced travelling, reduced consultation duration, and increased consultation frequency with the patient would contribute to the predicted increase. In Survey 2021, 88% of those with experiences of VCs perceived an increased access to healthcare (Table 2). Compliance with dietetic treatment was expected to be unchanged (53%) or decreased (25%), and the quality of the relationship with their patients was also expected to be unchanged (46%) or decreased (43%) by the RDs in 2016, compared to FTFCs. In 2021, the treatment quality was either perceived to be unchanged (55%) or decreased (31%) when VCs were compared to FTFCs. Relationship quality was seen to be unchanged by 52%, but decreased by 37%.

**Figure 4:**
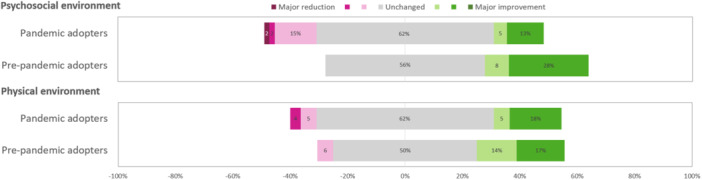
Perceptions of changes in the working environment when video consultations are compared to face‐to‐face consultations, rated on a 7‐graded likert scale from Major reduction to Major improvement, by dietitians with experience of video consultations separated into pre‐pandemic adopters and pandemic adopters, in Survey 2021 (*n* = 73).

The perceived effect of VCs on the RD's physical and psychosocial working environment was unchanged to positive compared to FTFCs in Survey 2021 (Table 2). Pre‐pandemic adopters reported an improvement in the psychosocial working environment compared to pandemic adopters (*p* = 0.018) (Figure 4).

## 4 Discussion

This study investigated the use and perceptions of VC appropriateness in clinical practice among Swedish RDs before and after the onset of the COVID‐19 pandemic in March 2020. The study showed an over 300% increase in the use of VCs as a consultation modality between the RDs participating in 2016 and 2021, respectively. Registered Dietitians in both years believed VCs increased access to nutritional care, with the therapeutic relationship remaining unchanged to slightly decreased. They considered VCs suitable for individuals living far from nutritional care. However, consultations requiring language interpretation were considered less suitable for VCs by a majority of the RDs in 2021.

The sharp increase in VC use among RDs aligns with increases in Italy (300% [40]) and the US (100% [41]) after pandemic onset. Telehealth ensured consistent nutritional care during the pandemic [39, 40, 41], which may contribute to the positive attitudes towards VCs from RDs in 2021. This rise is likely to have been enabled by increased *access* to VCs before and during COVID‐19, as suggested by Levesque et al.'s framework [22]. With telehealth applications implemented in most Swedish healthcare regions [27], VCs *availability* increased. Registered Dietitians' *acceptance* of VCs grew as it was a secure alternative to FTFCs and was positively received by the RD community [39, 40, 41]. Increases in *availability* and *acceptance* might have made patients more aware of VCs, further enhancing the ability to *reach* and *seek* care. Overall, the increased VC *access*, maintained *access* to healthcare during COVID‐19, confirming rapid telehealth implementations during crises [14, 15, 16]. However, previous crisis‐induced implementations often faded out once the crisis was over. The higher VC use in primary care compared to hospital care by RDs aligns with increases in VC use in Swedish primary care generally [29], perhaps due to a perceived need for improved access [47]. The RDs' assumption of increased accessibility to care with VCs for patients with reduced access to FTFCs aligns with previous research for rural areas [4, 6]. The COVID‐19 period, with global enhancements in the implementation of telehealth [1], combined with infrastructure developments, rising digital literacy, and increased sustainability awareness [2], might be the tipping point for broad, permanent VC implementation beyond rural areas due to enhanced acceptability.

Nevertheless, the accessibility of telehealth remains limited for certain groups, such as individuals with disabilities, low socioeconomic status, and those needing interpretational support [3, 18]. The RDs in 2021 indicated consultations with individuals needing interpreters as least suitable for VCs. Findings from Australia conclude restrained access to telehealth for this group although high trust in telehealth was found from the patients [19]. From the perspective of Levesque's conceptual framework [22], lower *approachability* for VCs regarding individuals needing interpreter services restricts *access* to VCs, perhaps because RDs perceived VCs as less *available* or less *appropriate* to this group. The WHO report on global standards for telehealth accessibility recommends better access for individuals with disabilities, such as requirements for deaf and hard‐of‐hearing individuals [3]. Similar recommendations seem to be needed to increase VC access for those needing language interpreters. Increased access to VCs for a broad range of individuals can enhance healthcare equality and minimise the risk of restricted access to healthcare during a crisis.

The RDs' assumption in both surveys about unchanged to slightly reduced therapeutic relationship quality between RDs and patients seems connected to their similar assumption about the quality of care in 2021. Previous research shows lower quality scores for VCs compared to FTFCs [21]. A central focus of the nutritional care process is the interaction between RDs and patients, which can form a relationship [36]. This relationship facilitates the quality of care and positively affects treatment outcomes. The high rate of RD‐driven consultation modality choices reported in 2021 indicates that the RDs' preconceptions of consultation modalities play a significant role in these choices. From Levesque's conceptual framework perspective [22], *access* to care by VCs is determined by the RD's assumption regarding its *appropriateness*, affecting the *approachability* of VCs and patients' ability to *perceive* VC‐care. The greater use of VCs by pre‐pandemic adopters in our study is most likely connected to the longer time period of implementation, larger catchment areas, and higher perceived psychosocial benefits, and affecting their perception of *appropriateness*. The difficulty in anthropometric measurements and relational development mentioned by RDs in both surveys aligns with previously studied barriers [21, 31, 37, 41]. However, studies have also shown alternative ways of using VCs for assessments, such as asking specified questions [33, 37, 48]. The RDs perception of the therapeutic relationship with the patient shifted positively by 10% between survey 2016 to survey 2021, from decreased to unchanged with VCs, perhaps indicating a tendency to shift in perception. The significant difference in RDs’ assumptions about VC suitability for follow‐up versus first‐time consultations in 2016 is supported by other studies, which find telehealth most suitable for follow‐ups because there is less need for relational establishment and measurements [20, 49]. Our results from 2021 indicate that VCs were evenly used for first‐time and follow‐up consultations, compared to FTFCs and TCs with more distinct partitions between them. This result, combined with the suitability rating for long distances by RDs in 2021, suggests that quality lapses identified with VCs are either compensated for or overlooked when access is considered more important [50]. However, the relatively low frequency of VCs, even under COVID‐19 restrictions in 2021, indicates that further improvements regarding appropriateness factors for access to healthcare via VCs are needed, such as standards for nutritional assessments using VCs [31].

Our summarized findings interpreted from Levesque's conceptual framework perspective [22] were that increased availability of VCs was observed because of greater access and acceptance. This increased access to VCs helped maintain healthcare services during restrictions. Enhanced availability and acceptance improved patient ability to perceive and seek VC‐care. However, perceptions of lower availability or appropriateness with VCs most likely impacted the approachability of VCs negatively, particularly for individuals needing interpreter services. Access to care via VCs is influenced by RDs assumptions regarding its appropriateness, which in turn affects the approachability of VCs and access to healthcare by VCs.

Investigating VC use and perceptions before and after the COVID‐19 onset was a key strength of this study as it captured data before the pandemic restrictions rather than only in retrospect. However, given the low participation rate and nonprobability sampling method [44], no general assumptions for the whole RD population in Sweden can be made. The context of consultation modalities in clinical settings was severely altered between 2016 and 2021, inducing a need for the survey questions in 2021 to be reformulated. Thereby, comparisons between the surveys were difficult to make. The participating RDs likely had an interest in VCs, visible through their engagement with answers to the open‐ended questions in 2016, and the high positivity for VCs in 2021. Despite the survey participation indirectly requiring membership in the DRF, or the use of selected social media groups, most RDs in Sweden are believed to have had the opportunity to participate. Both surveys received answers from most healthcare regions, and proportionately more from regions with higher inhabitant density. Overall, the characteristics of the participants correspond well with the RD population in Sweden, except that the RDs were slightly older than the population in 2016 [23, 51]. All considered, we believe that our study gives insight and understanding of RD's perceptions and the use of VCs in Sweden.

## 5 Conclusion

In conclusion, this study indicates a broader implementation of VCs among Swedish RDs after the onset of COVID‐19, compared to pre‐pandemic times. However, barriers to VCs must be overcome for individuals in need of a language interpreter, to reduce inequalities. Implications for quality and therapeutic bond enhancement techniques, to further increase the frequency of VC use, were also observed. The RDs' perceptions of appropriateness for VCs affect access to healthcare. Additional research is needed to follow up on VC use after pandemic restrictions have been lifted, and to further study concerns and conditions important for effective VC implementation and the quality of care in that modality.

## Author Contributions

Survey 2016 was drafted by S.P., P.R., and A.E.L. Survey 2021 was drafted by S.P., P.R., C.O., and A.E.L. S.P. collected and analysed the data together with P.R. S.P. drafted the manuscript together with P.R. and additional critical revision from A.E.L. and C.O. Figures and tables were created by P.R. together with S.P. The final draft was approved by all authors.

## Ethics Statement

The study follows the Declaration of Helsinki and has been approved by the Swedish Ethical Review Authority (dnr 2015/330‐31).

## Conflicts of Interest

The authors declare no conflicts of interest.

## Peer Review

The peer review history for this article is available at https://www.webofscience.com/api/gateway/wos/peer-review/10.1111/jhn.70080.

## Data Availability

The data that support the findings of this study are available on request from the corresponding author. The data are not publicly available due to privacy or ethical restrictions.
